# Causal effects of socioeconomic traits on frailty: a Mendelian randomization study

**DOI:** 10.3389/fmed.2024.1344217

**Published:** 2024-07-12

**Authors:** Jian Huang, Ying Gui, Jing Wu, Yubo Xie

**Affiliations:** ^1^Clinical Laboratory Center, The First Affiliated Hospital of Guangxi Medical University, Nanning, China; ^2^Department of Anesthesiology, The First Affiliated Hospital of Guangxi Medical University, Nanning, China; ^3^Guangxi Key Laboratory of Enhanced Recovery After Surgery for Gastrointestinal Cancer, The First Affiliated Hospital of Guangxi Medical University, Nanning, China

**Keywords:** Mendelian randomization, socioeconomic status, frailty, summary statistics, causal relationship

## Abstract

**Background:**

The relationship between socioeconomic status and frailty has been extensively investigated in the literature, but it remains unclear whether a causal relationship exists. Our goal is to evaluate the causal relationship between six socioeconomic traits and the frailty index using summary-level data for single nucleotide polymorphisms from large genome-wide association studies with individuals of European ancestry.

**Methods:**

A two-sample MR was performed. We applied the inverse variance weighted (IVW) method for the primary estimate, with sensitivity analyses conducted using alternative MR methods to evaluate the robustness of the findings. A subsequent multivariable MR was undertaken to adjust for the effects of body mass index (BMI). Finally, the MR Steiger directionality test was performed to confirm the causal direction.

**Results:**

The IVW MR analysis revealed significant associations between various socioeconomic factors and the frailty index. Specifically, genetically predicated age completed full time education (β = −0.477, 95% confidence interval [CI]: −0.634 to −0.319) and average total household income before tax (β = −0.321, 95% CI: −0.410 to −0.232) were negatively associated with the frailty index. On the other hand, genetically predicted job involves heavy manual or physical work (β = 0.298, 95% CI: 0.113 to 0.484), job involves mainly walking or standing (β = 0.179, 95% CI: 0.013 to 0.345), Townsend deprivation index at recruitment (β = 0.535, 95% CI: 0.285 to 0.785), and social isolation/loneliness (β = 1.344, 95% CI: 0.834 to 1.853) were positively associated with the frailty index. Sensitivity analysis using other MR methods and multivariable MR analysis adjusting for BMI yielded stable results. The MR Steiger directionality test confirmed the causal direction.

**Conclusion:**

Our findings highlight the importance of socioeconomic factors in affecting frailty risk. Future research should focus on unraveling the pathways through which these socioeconomic factors exert their effects on frailty, with the ultimate goal of developing targeted strategies to mitigate the risk of frailty.

## Introduction

Frailty is a significant indicator of aging, characterized by a decrease in functional capacity and heightened susceptibility to illness (1). It is estimated that the prevalence of frailty in the general population is around 14% (2). Due to the aging population, its incidence continues to rise. Frailty is strongly associated with various unfavorable health outcomes, including depression, dementia, physical disability, hospitalization, reduced quality of life, and increased mortality (1, 3, 4). It is widely acknowledged that frailty is one of the gravest public health concerns of the 21st century. Therefore, knowledge of the factors that contribute to frailty is vital to determine the specific population groups who may benefit from interventions aimed at preventing frailty.

Over the past two decades, a number of epidemiological studies have evaluated the association between socioeconomic status and frailty. Aspects related to socioeconomic status in this context include education, job, income, and social activity. Many researchers have reported that educational attainment, job-related characteristics, economic status, and social isolation are associated with frailty (5–10). In their study based on nationally representative samples of adults aged 50 years and older, Harttgen and colleagues observed that individuals with lower levels of education and income displayed a higher incidence of frailty (5). Using a cross-sectional study design, de Amorim and coworkers found that the prevalence of frailty was significantly higher among elderly individuals with low levels of education, who worked in unhealthy/dangerous environments and whose job primarily involved physical labor (6). Moreover, the study by Tatoli et al. (10) reported that socioeconomic status may impact health outcomes among the elderly population by influencing their diet and eating habits. A recent systematic review summarized 21 studies covering a wide range of geographic population and emphasize on the importance of socioeconomic status on frailty development (11). However, different results were also reported. The study by Delbari et al. (12), which focused on a random sample of adults aged 60 years and above from five Southwest cities in Iran, did not demonstrate any significant link between education attainment, income, and frailty. Goodyer et al. also did not find an association between socioeconomic position and frailty among elderly people seen by the specialty frailty service (13). It should be noted that inconsistent findings were evident in the literature. In addition, although some authors suggested a link between socioeconomic status and frailty, causality could not be inferred in their studies due to the limitations of conventional epidemiological methods. These limitations primarily stemmed from the inability to control for possible confounding variables and the potential for bias in study design or data collection. Additionally, the lack of randomization in observational studies introduced the possibility of unmeasured or residual confounding, further complicating the interpretation of findings.

Mendelian randomization (MR) is a powerful causal inference technique for probing the causal association between modifiable exposures and economic, social, and health outcomes (14). When specific assumptions are satisfied, the utilization of genetic variants as instrumental variables can help mitigate concerns regarding reverse causation and confounding biases, thereby strengthening inferences drawn from observational studies. The aim of this study was to use MR analyses to estimate the causal effects of socioeconomic traits, including genetically predicted age completed full time education, job involves heavy manual or physical work, job involves mainly walking or standing, average total household income before tax, Townsend deprivation index at recruitment, and social isolation/loneliness on frailty.

## Methods

### Ethical approval

This MR study was exempt from Institutional Review Board ethical approval due to the utilization of publicly accessible de-identified summary statistics.

### Study design

**Figure 1:**
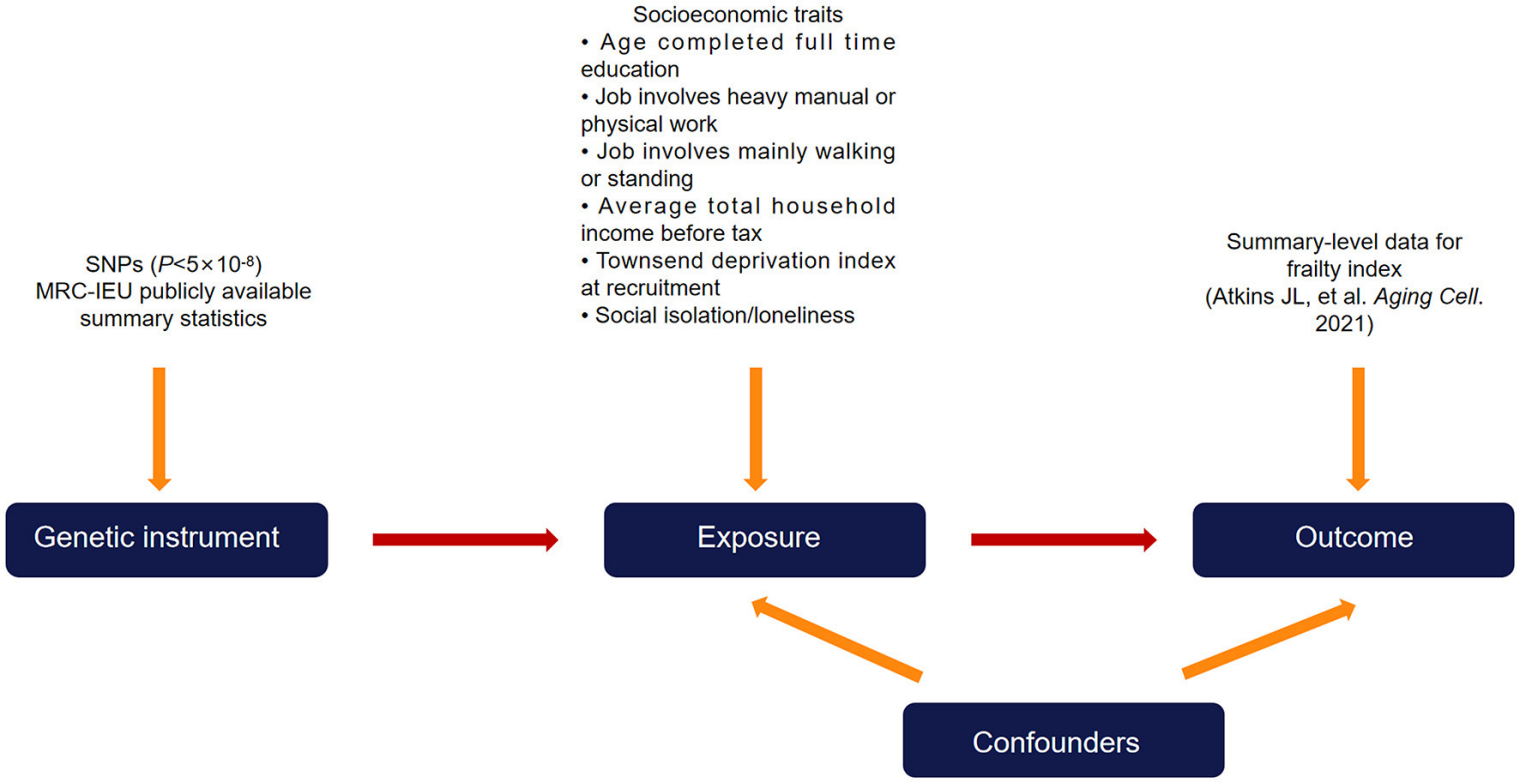
Diagram of the two-sample Mendelian randomization study design. Single nucleotide polymorphisms that are significantly associated with the exposure (socioeconomic traits) from publicly available summary-level data were used as instrumental variables. The instrumental variables affect the outcome (frailty index) exclusively through the exposure and not through other pathways.

Utilizing summary-level genetic data from large-sample genomewide association studies (GWASs), we implemented a two sample MR study design in the present study (Figure 1). There exist three essential assumptions that need to be fulfilled in order to guarantee the validity of an MR study, namely: (a) the genetic variants used as instruments ought to exhibit a strong correlation with the targeted risk factor; (b) confounding variables do not affect the relationship between the genetic variants and the outcome; and (c) the genetic variants only affect the outcome through the risk factor.

### Genetic data on exposures

Single nucleotide polymorphisms (SNPs) were extracted from summary-level statistics for age completed full time education (*N* = 307,897), job involves heavy manual or physical work (*N* = 263,615), job involves mainly walking or standing (*N* = 263,556), average total household income before tax (*N* = 397,751), townsend deprivation index at recruitment (*N* = 462,464), and social isolation/loneliness (*N* = 455,364) from the Medical Research Council Integrative Epidemiology Unit (MRC-IEU) OpenGWAS data infrastructure (15). The GWASs on these exposures were performed in UK Biobank participants. Within the UK Biobank cohort, more than 500,000 individuals were recruited across Scotland, England, and Wales from 2006 to 2010, ranging in age from 40 to 69 years. To mitigate any potential influence from population stratification, we exclusively selected individuals of European ancestry for inclusion in the exposure datasets. For each exposure, the threshold for instrumental SNP extraction was set at *P* < 5 × 10^−8^ (genome-wide significance). The estimation of linkage disequilibrium between these SNPs was conducted utilizing the European subset of 1,000 Genomes (16). We did not consider SNPs with an estimated linkage disequilibrium greater than an r^2^ value of 0.001. Palindromic SNPs were not included in the MR analyses. The F-statistics for instrument strength were calculated in our study as previously described (17); generally, a value >10 for this statistic indicates adequate strength. Table 1 shows the details on the summary statistics used for MR analyses. Supplementary Table 1 presents detailed information on the six socioeconomic traits.

**Table 1:** The GWAS datasets used in the present Mendelian randomization study.

**Year**	**Author**	**Trait**	**Sample size**	**Sex**	**Population**	**GWAS identifier**	**Consortium**
2018	Ben Elsworth	Age completed full time education	307,897	Males and females	European	ukb-b-6134	MRC-IEU
2018	Ben Elsworth	Job involves heavy manual or physical work	263,615	Males and females	European	ukb-b-2002	MRC-IEU
2018	Ben Elsworth	Job involves mainly walking or standing	263,556	Males and females	European	ukb-b-4461	MRC-IEU
2018	Ben Elsworth	Average total household income before tax	397,751	Males and females	European	ukb-b-7408	MRC-IEU
2018	Ben Elsworth	Townsend deprivation index at recruitment	462,464	Males and females	European	ukb-b-10011	MRC-IEU
2018	Ben Elsworth	Social isolation/loneliness	455,364	Males and females	European	ukb-b-8476	MRC-IEU
2018	Yengo L	Body mass index	681,275	Males and females	European	ieu-b-40	GIANT
2021	Atkins JL	Frailty index	175,226	Males and females	European	ebi-a-GCST90020053	NA

GIANT, Genetic Investigation of ANthropometric Traits; GWAS, genome-wide association studies; MRC-IEU, Medical Research Council-Integrative Epidemiology Unit; NA, not applicable.

### Genetic data on frailty

A recent meta-analysis of genome-wide association studies conducted in the UK Biobank and Swedish TwinGene (18), involving 175,226 participants of European descent, yielded summary statistics for frailty measured using the frailty index. In the UK Biobank study, the samples had an age range of 60 to 70 years, with a mean age of 64.1 years and a standard deviation (SD) of 2.8. The participants in the Swedish TwinGene study, on the other hand, were aged between 41 and 87 years, with a mean age of 58.3 years and a SD of 7.9. The frailty index, well-validated in the literature, is widely utilized as a measure of frailty in clinical practice (19). In order to calculate the frailty index, the researchers utilized self-reported data on symptoms, disabilities, and diagnosed diseases. This was done using 49 items for UK Biobank and 44 items for TwinGene (18). More detailed information can be referred to the original study (18).

### Statistical analyses

The primary analysis focused on evaluating the association between frailty and socioeconomic traits through the use of an inverse variance weighted (IVW) two-sample MR technique (20). For each exposure, the contribution of each instrumental SNP to the risk of frailty was evaluated through a weighting process, considering its effect on the exposure using the Wald ratio method (21). By employing a random effect inverse-variance meta-analysis, these individual estimates obtained from MR were pooled together. Despite the IVW method's ability to provide the most accurate causal estimations, it can be affected by pleiotropy and outlying SNPs (21). Therefore, by employing alternative MR approaches, we were able to generate unbiased estimates, even when dealing with potentially invalid genetic instruments. We considered four alternative MR techniques, including MR-Egger, weighted median, maximum likelihood, and MR-pleiotropy residual sum and outlier (MR-PRESSO). Moreover, by implementing leave-one-out analysis, each SNP was sequentially omitted to observe the individual influences on the causal association determined by the IVW method. To evaluate the existence of horizontal pleiotropy, we relied on the *P*-value obtained from the intercept test in MR-Egger regression (22). The Cochran's Q test was employed to evaluate the heterogeneity among MR estimates generated by different SNPs (23). The application of Steiger directionality test allowed us to evaluate the direction of causality (24). By comparing the connection of the instrumental variables with both the exposure and the outcome, the Steiger directionality test evaluates the phenotypic variance accounted for by instrumental SNPs to verify the correctness of the assumed causal direction (24). Previous observational studies and MR investigations revealed that BMI may be an important risk factor for frailty (25–28). The MR study by Zhang et al. (27) found that genetically predicted one-SD increase in BMI was associated with higher risk of frailty (β = 0.118. 95% CI: 0.079 to 0.158, *P* = 4.4 × 10^−9^). Another MR study by Gu and colleagues also demonstrated that an increase in BMI by one SD lead to an increased probability of frailty (28). Thus, besides univariable MR, multivariable MR was conducted controlling for BMI (GWAS identifier: ieu-b-40) (Table 1).

The “TwoSampleMR” and “MR-PRESSO” packages were employed for all the analyses in R version 4.1.0. These packages facilitates data formatting, harmonization, and utilization of MR techniques in a partially automated way (15, 24).

## Results

The number of instrument SNPs predicting age completed full time education, job involves heavy manual or physical work, job involves mainly walking or standing, average total household income before tax, Townsend deprivation index at recruitment, and social isolation/loneliness were 37, 24, 15, 42, 17, and 16, respectively. The characteristic features of the instrument SNPs for each exposure, including beta values, standard error, effect allele, other allele, rsID, F-statistics, etc., are represented in Supplementary Tables 2–7. Each of the instrumental variables displayed an F-statistic of greater than or equal to 16, surpassing the standard cutoff of >10, thereby suggesting a strong instrumental strength.

**Figure 2:**
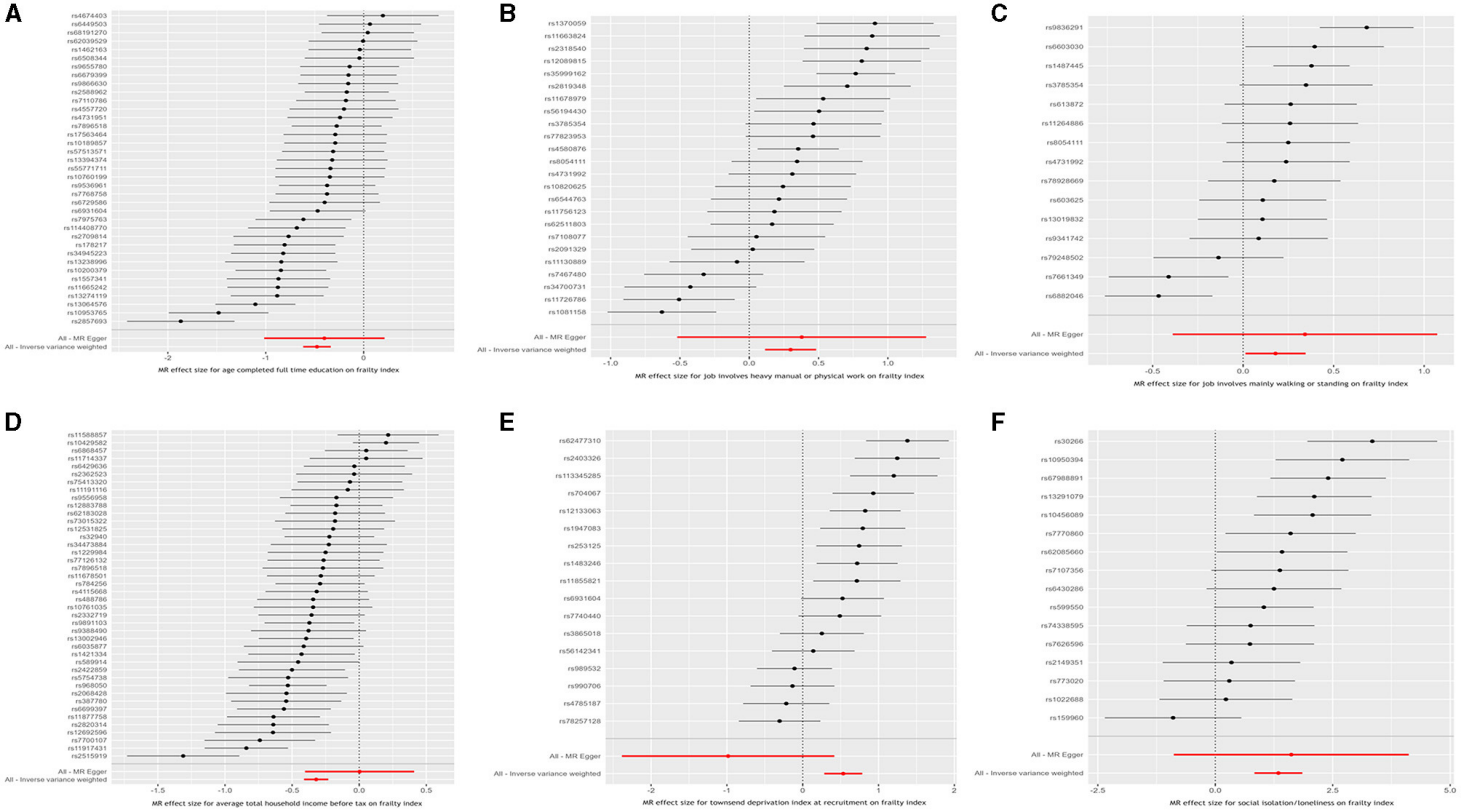
Inverse variance weighted Mendelian randomization estimates for the causal relationship between six socioeconomic traits and the frailty index. **(A)** Age completed full time education; **(B)** Job involves heavy manual or physical work; **(C)** Job involves mainly walking or standing; **(D)** Average total household income before tax; **(E)** Townsend deprivation index at recruitment; and **(F)** Social isolation/loneliness.

Results from the MR analyses are visualized in Figure 2, while Table 2 presents the corresponding data. In univariable IVW MR analysis, genetically predicated age completed full time education (β = −0.477, 95% confidence interval [CI]: −0.634 to −0.319, *P* = 2.995 × 10^−11^) and average total household income before tax (β = −0.321, 95% CI: −0.410 to −0.232, *P* = 2.810 × 10^−12^) were negatively associated with the frailty index, while genetically predicted job involves heavy manual or physical work (β = 0.298, 95% CI: 0.113 to 0.484, *P* = 1.490 × 10^−3^), job involves mainly walking or standing (β = 0.179, 95% CI: 0.013 to 0.345, *P* = 3.452 × 10^−2^), Townsend deprivation index at recruitment (β = 0.535, 95% CI: 0.285 to 0.785, *P* = 2.715 × 10^−5^), and social isolation/loneliness (β = 1.344, 95% CI: 0.834 to 1.853, *P* = 2.367 × 10^−7^) were positively associated with the frailty index. Across a range of sensitivity analyses, including those utilizing weighted median, maximum likelihood, and MR-PRESSO methods, the MR estimates observed remained largely consistent (Table 2). There was no evidence of possible pleiotropy based on the intercept in the MR-Egger regression except for the analysis of Townsend deprivation index at recruitment (Table 3). However, MR-PRESSO did not detect any outlying SNPs for Townsend deprivation index at recruitment. Heterogeneity was observed in the analyses for all exposures (Table 3).

**Table 2:** Results of Mendelian randomization analysis for the causal effects of socioeconomic traits on the frailty index.

**Traits**	**Mendelian randomization approach**	**Instrument SNPs' number**	**The frailty index**		
			**Beta (95% CI)**	**SE**	***P*-value**
Age completed full time education	IVW	37	−0.477 (−0.634 to −0.319)	0.072	2.995 × 10^−11^
	Weighted median	37	−0.313 (−0.450 to −0.176)	0.070	7.533 × 10^−6^
	MR-PRESSO (corrected)	35	−0.413 (−0.527 to −0.299)	0.058	2.938 × 10^−8^
	Maximum likelihood	37	−0.465 (−0.555 to −0.375)	0.046	4.107 × 10^−24^
	MR-Egger	37	−0.401 (−1.016 to 0.213)	0.314	2.092 × 10^−1^
Job involves heavy manual or physical work	IVW	24	0.298 (0.113 to 0.484)	0.094	1.490 × 10^−3^
	Weighted median	24	0.371 (0.177 to 0.504)	0.083	4.137 × 10^−5^
	MR-PRESSO (corrected)	20	0.317 (0.161 to 0.472)	0.079	7.734 × 10^−4^
	Maximum likelihood	24	0.308 (0.212 to 0.404)	0.049	2.919 × 10^−10^
	MR-Egger	24	0.379 (−0.520 to 1.278)	0.458	4.171 × 10^−1^
Job involves mainly walking or standing	IVW	15	0.179 (0.013 to 0.345)	0.085	3.452 × 10^−2^
	Weighted median	15	0.245 (0.111 to 0.379)	0.068	3.234 × 10^−4^
	MR-PRESSO (corrected)	12	0.228 (0.142 to 0.314)	0.044	2.922 × 10^−4^
	Maximum likelihood	15	0.191 (0.101 to 0.281)	0.046	2.697 × 10^−5^
	MR-Egger	15	0.342 (−0.390 to 1.075)	0.373	3.758 × 10^−1^
Average total household income before tax	IVW	42	−0.321 (−0.410 to −0.232)	0.046	2.810 × 10^−12^
Weighted median	42	−0.288 (−0.379 to −0.197)	0.046	5.748 × 10^−10^
MR-PRESSO (corrected)	39	−0.311 (−0.380 to −0.242)	0.035	9.876 × 10^−11^
Maximum likelihood	42	−0.316 (−0.378 to −0.254)	0.032	1.314 × 10^−23^
MR-Egger	42	0.003 (−0.403 to 0.409)	0.207	9.886 × 10^−1^
Townsend deprivation index at recruitment	IVW	17	0.535 (0.285 to 0.785)	0.127	2.715 × 10^−5^
	Weighted median	17	0.522 (0.274 to 0.769)	0.126	3.609 × 10^−5^
	MR-PRESSO (raw)	17	0.535 (0.285 to 0.785)	0.127	6.840 × 10^−4^
	Maximum likelihood	17	0.557 (0.413 to 0.701)	0.073	3.459 × 10^−14^
	MR-Egger	17	−0.984 (−2.384 to 0.417)	0.715	1.889 × 10^−1^
Social isolation/loneliness	IVW	16	1.344 (0.834 to 1.853)	0.260	2.367 × 10^−7^
	Weighted median	16	1.202 (0.646 to 1.759)	0.284	2.306 × 10^−8^
	MR-PRESSO (corrected)	15	1.470 (1.009 to 1.930)	0.235	2.103 × 10^−5^
	Maximum likelihood	16	1.415 (1.051 to 1.779)	0.186	2.410 × 10^−14^
	MR-Egger	16	1.618 (−0.883 to 4.118)	1.276	2.255 × 10^−1^

SNP, single nucleotide polymorphism; IVW, inverse variance weighted; CI, confidence interval; SE, standard error; MR-Egger, Mendelian randomization-Egger; MR-PRESSO, Mendelian randomization pleiotropy residual sum and outlier.

**Table 3:** Evaluation of heterogeneity and pleiotropy of the Mendelian randomization analyses.

**Exposure**	**Instrument SNPs' number**	**Cochran's Q**	**Egger's intercept**	***P*-value of intercept**	**Number of outliers detected by MR-PRESSO**
Age completed full time education	37	100.059	−0.001	0.806	2
Job involves heavy manual or physical work	24	100.565	−0.001	0.859	4
Job involves mainly walking or standing	15	54.542	−0.004	0.661	3
Average total household income before tax	42	96.306	−0.006	0.117	3
Townsend deprivation index at recruitment	17	57.573	0.021	0.048	0
Social isolation/loneliness	16	34.686	−0.002	0.829	1

MR-PRESSO, MR-pleiotropy residual sum and outlier.

In multivariable MR analysis adjusting for genetically predicted BMI, the negative associations of genetically predicted age completed full time education (β = −0.374, 95% CI: −0.493 to −0.255, *P* = 5.379 × 10^−10^; 5 SNPs) and average total household income before tax (β = −0.258, 95% CI: −0.343 to −0.173, *P* = 3.018 × 10^−9^, 11 SNPs) with the frailty index and the positive associations of genetically predicted job involves heavy manual or physical work (β = 0.310, 95% CI: 0.207 to 0.413, *P* = 4.375 × 10^−9^, 4 SNPs), job involves mainly walking or standing (β = 0.202, 95% CI: 0.125 to 0.279, *P* = 3.410 × 10^−7^, 4 SNPs), Townsend deprivation index at recruitment (β = 0.360, 95% CI: 0.228 to 0.491, *P* = 8.335 × 10^−8^, 2 SNPs), and social isolation/loneliness (β = 1.615, 95% CI: 1.292 to 1.938, *P* = 1.230 × 10^−22^, 2 SNPs) with the frailty index remained.

**Figure 3:**
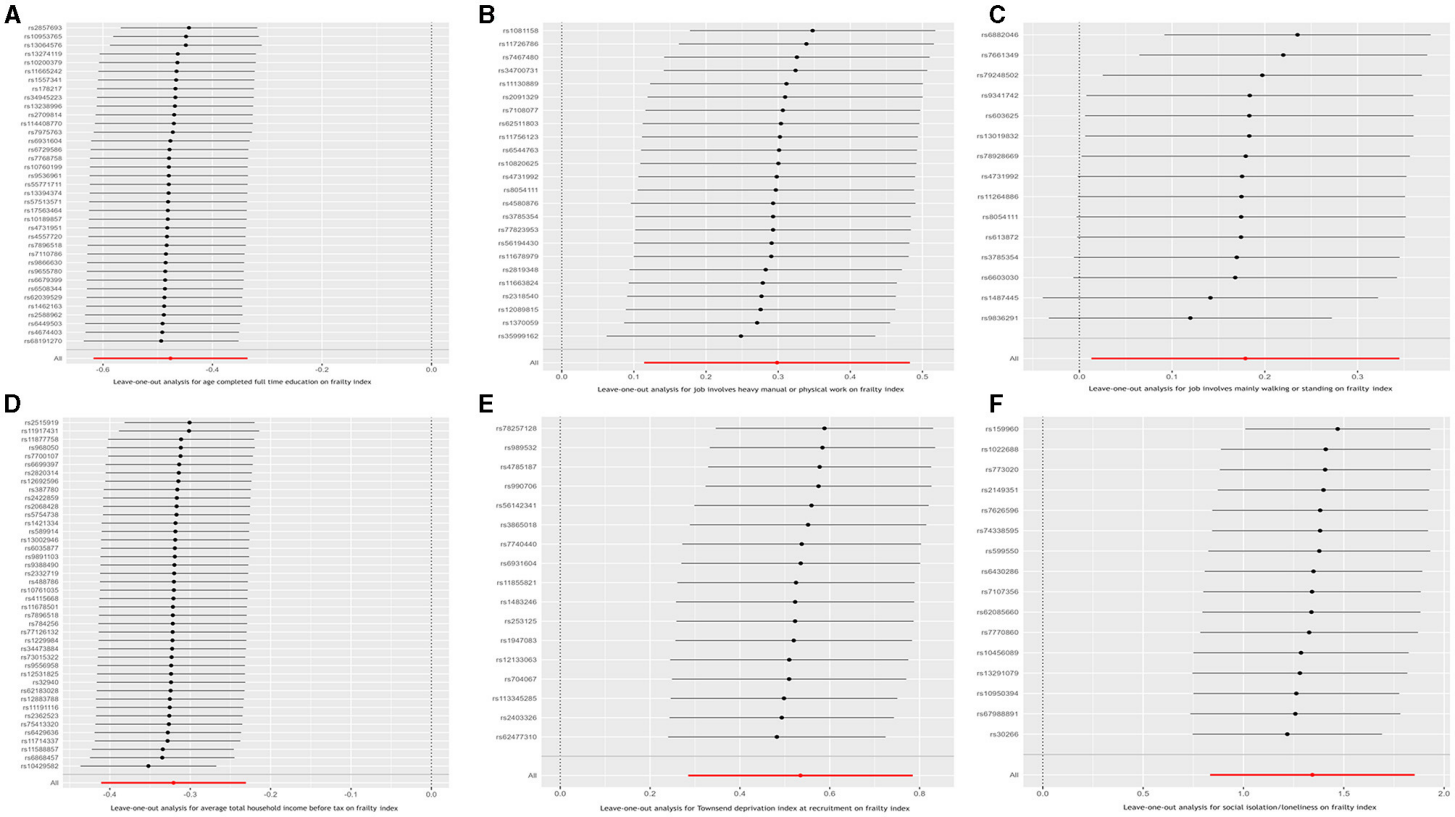
Leave-one-out analysis for the causal relationship between six socioeconomic traits and the frailty index. **(A)** Age completed full time education; **(B)** Job involves heavy manual or physical work; **(C)** Job involves mainly walking or standing; **(D)** Average total household income before tax; **(E)** Townsend deprivation index at recruitment; and **(F)** Social isolation/loneliness.

Finally, to ensure the direction of causality, we performed the MR Steiger directionality test (24). Table 4 shows the results. The direction of the socioeconomic traits' causal effects was confirmed. Finally, consistency in MR estimates was observed across the leave-one-out analyses (Figure 3).

**Table 4:** Results of the MR Steiger directionality test checking causality direction.

**Exposure**	**Outcome**	**R^2^ for exposure**	**R^2^ for outcome**	**Correct causal direction**	**Steiger *P*-value**
Age completed full time education	Frailty	4.686 × 10^−3^	1.287 × 10^−3^	True	0
Job involves heavy manual or physical work	Frailty	3.575 × 10^−3^	8.075 × 10^−4^	True	0
Job involves mainly walking or standing	Frailty	2.579 × 10^−3^	4.867 × 10^−4^	True	0
Average total household income before tax	Frailty	4.659 × 10^−3^	1.258 × 10^−3^	True	0
Townsend deprivation index at recruitment	Frailty	1.272 × 10^−3^	6.827 × 10^−4^	True	6.71 × 10^−4^
Social isolation/loneliness	Frailty	1.260 × 10^−3^	5.463 × 10^−4^	True	1.58 × 10^−5^

MR, Mendelian randomization.

## Discussion

The causal associations between six socioeconomic traits and frailty risk were examined in this study using a MR framework. Our findings indicated that genetically predicted age completed full time education (educational attainment) and average total household income before tax were inversely associated with the frailty index, whereas genetically predicted job involves heavy manual or physical work, job involves mainly walking or standing, Townsend deprivation index at recruitment, and loneliness were positively associated with the frailty index. Furthermore, these causal associations remained significant in multivariable MR analyses accounting for BMI. The work provided evidence supporting a potential critical role of socioeconomic characteristics in the development of frailty.

Our findings are in concurrence with the results obtained from a recent MR study (18). Utilizing genetic risk scores for specific exposures and conducting sensitivity analyses to mitigate potential pleiotropy-related bias, Atkins et al. (18) unveiled compelling findings that implied a negative association between educational attainment and the frailty index among UK Biobank participants of European ancestry aged 60–70 years. However, their study only focused on educational attainment; other socioeconomic characteristics such as economic conditions and work status were not taken into account. On the other hand, observational and clinical trial evidence continues to mount, pointing to an association between income, occupational factors, and social isolation/loneliness and the risk of frailty. An observational study (29), using data from four waves of interviews conducted as part of the Chinese Longitudinal Healthy Longevity Survey between 2008 and 2018, recruited a total of 3,327 participants (mean age: 81.2; SD: 10.3). The study authors observed an overall frailty incidence of 37.5%. Additionally, they identified a significant association between higher household income and a reduced risk of frailty (*P* < 0.05). In another cross-sectional investigation comprising 301 European individuals aged 65 years or greater, findings revealed that elderly individuals with a yearly personal income above 4,500 euros had a lower likelihood of experiencing frailty (OR = 0.45, 95% CI: 0.25 to 0.83, *P* = 0.011) in comparison to those with an income below this threshold (30). The strong association between economic conditions and frailty was reaffirmed in a longitudinal investigation, which utilized data from the Survey on Health, Aging and Retirement in Europe (SHARE) and involved 12,002 participants aged 65 years and above (7). The study revealed that individuals who experienced deteriorating economic conditions, as indicated by reduced wealth and increased subjective deprivation, were at a heightened risk of developing frailty (7). In agreement with these studies, our MR analyses revealed that genetically predicted average total household income before tax had a protective impact on frailty, while genetically predicted townsend deprivation index at recruitment increased the risk of frailty. The Townsend deprivation index is a tool utilized to assess the degree of material deprivation in a population, taking into account factors such as unemployment, lack of car and home ownership, and household overcrowding (29). It is commonly employed in research studies to evaluate the level of poverty among participants. Our analyses contributed essential evidence that strengthened the assertion of a causal connection between economic conditions and frailty.

The relationship between occupational factors and frailty has also been a focal point in previous literature. In a cross-national survey of elderly individuals residing in five major cities in Latin America, Alvarado and colleagues identified a notable association between non-white-collar jobs and increased likelihood of frailty in both genders (31). Similarly, an American cohort including 1,857 non-institutionalized individuals aged 60 years and above revealed an elevated frailty risk in individuals with a job involving manual labor (adjusted OR = 2.24, 95% CI: 1.41 to 3.56) (32). Moreover, an observational study including 258 elderly workers ≥ 60 years reported that frailty was significantly associated with the workers whose job was predominantly physical (6). This positive association was confirmed in a recent complex analysis of the SHARE study including up to 8,411 adults whose average age was 72.4 years (SD 8.0), which observed that a physically demanding main lifetime occupation promoted the risk of frailty (*P* < 0.001) (33). However, there were also some studies that did not support an association between occupational factors and frailty (34). In accordance with the majority of studies, our analyses revealed strong MR associations of genetically predicted job involves heavy manual or physical work and job involves mainly walking or standing with frailty. These observed associations may be attributed to physical strain and chronic inflammation as potential underlying mechanisms. Jobs that involve strenuous manual labor or extended periods of walking/standing can place strain on the body through repetitive actions and excessive physical exertion. As time goes by, this can result in musculoskeletal injuries, joint degeneration, and the deterioration of physical health, thereby augmenting the vulnerability to frailty (35, 36). In addition, intense physical work and prolonged standing/walking may contribute to chronic inflammation in the body (37, 38). Persistent inflammation has been associated with a range of adverse health outcomes, including frailty. The body's inflammatory response to physical stressors could potentially accelerate the aging process and increase the risk of frailty.

Social isolation/loneliness has recently gained significant attention from researchers as a potential risk factor for frailty. Based on the data collected from the English Longitudinal Study of Aging (ELSA) spanning from 2004 to 2017 and involving over 9,000 participants aged 50 years and above, Davies et al. (9) conducted a longitudinal study revealing a significant association between heightened levels of social isolation or loneliness and an elevated frailty index score (beta = 0.006, 95% CI: 0.006 to 0.007, *P* < 0.0001). This positive association was also observed in cross-sectional or cohort studies performed in other countries, including Italy, Singapore, China, and Turkey (8, 39–41). Using a MR design, our analysis supported the causal effects of social isolation/loneliness on frailty. Social isolation/loneliness may impact frailty in several aspects. For example, social isolation or loneliness often results in a lack of social connections and reduced social support. This can further exacerbate an individual's susceptibility to physical and mental health issues associated with frailty (42). In addition, individuals lacking social interaction may be more prone to unhealthy dietary habits, lack of exercise, and inadequate sleep (43–45). The presence of these unhealthy lifestyle factors can contribute to the development of obesity, a decline in physical function, and the onset of chronic diseases, ultimately heightening the risk of frailty (46, 47). Given the detrimental influence of social isolation/loneliness, social support may be essential in preventing frailty among elderly individuals.

The implementation of a MR framework in our study represented a significant methodological strength, as it allowed us to draw causal inferences about the relationship between various socioeconomic traits and frailty. By incorporating six socioeconomic traits which reflected different aspects of socioeconomic status including education, income, occupation, and social activities, our analysis provided a comprehensive assessment of how socioeconomic status causally influenced the risk of frailty. Our findings underscored the importance of addressing socioeconomic inequalities to improve health outcomes such as frailty. For instance, our results suggested that higher educational attainment and income may protect against frailty, pointing to the potential benefits of investment in education and efforts to reduce income disparities. Moreover, the significance of social activities in preventing frailty highlighted the value of fostering strong community ties and supportive social networks. Overall, the causal insights gained from our MR study would have wide-ranging implications for public health policy, clinical practice, and individual decision-making. They emphasized the need for multidimensional strategies that considered the complex interplay of social and economic factors influencing frailty. By understanding these causal associations more deeply, we may develop targeted interventions and policies aimed at reducing the risk of frailty in individuals belonging to a low socioeconomic background. There is also a particular need to raise public awareness regarding the significant implications of socioeconomic status on frailty.

Another strength of our study is the utilization of both univariable and multivariable MR analyses and the consistency of results from them. Considering BMI as a covariate, we accounted for its effect using multivariable MR. We found that the associations of the socioeconomic traits with frailty remained significant in multivariable MR, indicating that their causal influence at frailty may be through pathways independent of BMI. Furthermore, the inclusion of only individuals of European ancestry in our analysis helped mitigate any bias arising from population stratification. After discussing the strengths of our study, it is important to acknowledge that there are several limitations. Firstly, while MR is a valuable statistical method for studying causality, it cannot fully replace high-quality randomized controlled trials (RCTs) in providing robust evidence of causal effects. Therefore, to elucidate the relationship between socioeconomic traits and frailty, high-quality RCTs are still necessary. Secondly, it is important to note that the study sample from the UK Biobank, which was the primary source for genetic association assessments in our analyses, may not accurately reflect broader populations, particularly those outside of European ancestry. Thirdly, the presence of pleiotropy poses a major threat to MR studies, implying that the instrumental variables affect outcome risk through alternative mechanisms unrelated to the exposure; but our MR estimates were unlikely to be biased by pleiotropy. Among the six socioeconomic traits we evaluated, MR-Egger intercept only indicated possible horizontal pleiotropy for Townsend deprivation index at recruitment. However, the MR-PRESSO analysis found no outliers for Townsend deprivation index at recruitment. Fourthly, the IVW method, as well as the supporting MR-PRESSO, weighted median, and maximum likelihood methods, indicated causal effects of socioeconomic traits on frailty, but the MR-Egger method yielded a non-significant estimate. It is worth noting that the MR-Egger method may yield different estimates due to its ability to adjust for potential pleiotropic effects. This adjustment can lead to wider confidence intervals, making the MR-Egger estimate more conservative and potentially explaining the discrepancy from other methods. While we acknowledged the inconsistency between the MR-Egger results and those obtained from other MR methods, it is worth noting that multiple complementary analyses supported causal effects of the socioeconomic traits on frailty. In addition, our multivariable MR analysis yielded stable results following adjustment for BMI.

In summary, our MR study yielded evidence indicating that genetically predicted age completed full time education and average total household income before tax conferred a protective effect against frailty. On the other hand, genetically predicted job involves heavy manual or physical work, job involves mainly walking or standing, Townsend deprivation index at recruitment, and social isolation/loneliness increased the risk of frailty. These findings underscore the importance of socioeconomic factors in causally influencing frailty risk and highlight potential avenues for intervention. Future studies should focus on unraveling the intricate mechanisms by which these socioeconomic factors impact frailty, aiming to ultimately develop targeted strategies to mitigate the risk of frailty across diverse populations.

## Data availability statement

In this Mendelian randomization study, we performed analyses using public datasets, which are accessible at this address: https://gwas.mrcieu.ac.uk/.

## Author contributions

JH: Conceptualization, Data curation, Formal analysis, Investigation, Methodology, Project administration, Resources, Software, Validation, Visualization, Writing—original draft, Writing—review & editing. YG: Investigation, Validation, Writing—review & editing. JW: Investigation, Validation, Writing—review & editing. YX: Funding acquisition, Supervision, Writing—review & editing.
