# Supplementary material for: Causal effects of socioeconomic traits on frailty: a Mendelian randomization study
Source: Front Med (Lausanne). 2024 Jul 12;11:1344217. doi: 10.3389/fmed.2024.1344217 (PMC11282504; doi:10.3389/fmed.2024.1344217)
Supplement: Supplementary file 1 [file Table_1.DOCX]

Supplementary Table 1 Characteristics of the datasets on the six socioeconomic traits.

| Traits | Dataset identifier in the IEU OpenGWAS database | Touchscreen question | Information | Answer options for touchscreen question |
| --- | --- | --- | --- | --- |
| Age completed full time education | ukb-b-6134 | At what age did you complete your continuous full time education? | The following checks were performed: (1) If answer < 5 then rejected; (2) If answer > Participants age then rejected; and (3) If answer > 40 then participant asked to confirm. | NA |
| Job involves heavy manual or physical work | ukb-b-2002 | Does your work involve heavy manual or physical work? | If the participant activated the Help button they were shown the message:  If you have more than one 'current job' then answer this question for your MAIN job only.  Physical work includes work that involves handling of heavy objects and use of heavy tools. | Never/rarely; Sometimes; Usually; Always; Do not know; Prefer not to answer. |
| Job involves mainly walking or standing | ukb-b-4461 | Does your work involve walking or standing for most of the time? | If the participant activated the Help button they were shown the message:  If you have more than one 'current job' then answer this question your MAIN job only. | Never/rarely; Sometimes; Usually; Always; Do not know; Prefer not to answer. |
| Average total household income before tax | ukb-b-7408 | What is the average total income before tax received by your HOUSEHOLD? | If the participant activated the Help button they were shown the message:  If you are unsure of annual household income here are the weekly and monthly equivalents:  Weekly: less than £346; £346 to £576; £577 to £999; £1000 to £1,923; greater than £1,923  Monthly: less than £1,500; £1,500 to £2,499; £2,500 to £4,333; £4,334 to £8,333; greater than £8,333 | Less than 18,000; 18,000 to 30,999; 31,000 to 51,999; 52,000 to 100,000; Greater than 100,000; Do not know; Prefer not to answer. |
| Townsend deprivation index at recruitment | ukb-b-10011 | NA | Townsend deprivation index calculated immediately prior to participant joining UK Biobank. Based on the preceding national census output areas. Each participant is assigned a score corresponding to the output area in which their postcode is located. | NA |
| Social isolation/loneliness | ukb-b-8476 | Do you often feel lonely? | If the participant activated the Help button they were shown the message:  Work through these questions quickly and do not think about the exact meaning of the question. | Yes; No; Do not know; Prefer not to answer. |

NA, not applicable.
