# Supplementary material for: Causal effects of socioeconomic traits on frailty: a Mendelian randomization study
Source: Front Med (Lausanne). 2024 Jul 12;11:1344217. doi: 10.3389/fmed.2024.1344217 (PMC11282504; doi:10.3389/fmed.2024.1344217)
Supplement: Supplementary file 2 [file Table_2.DOCX]

Supplementary Table 2 Characteristics of the instrument SNPs for age completed full time education.

| **SNP** | **Chr** | **Position** | **EA** | **OA** | **Exposure effect** |  |  | **F-statistic** |
| --- | --- | --- | --- | --- | --- | --- | --- | --- |
|  |  |  |  |  | **β** | **SE** | ***P*** |  |
| rs10189857 | 2 | 60713235 | G | A | -0.012 | 0.002 | 2.70E-09 | 36 |
| rs10200379 | 2 | 144183008 | A | C | 0.014 | 0.002 | 1.60E-11 | 49 |
| rs10760199 | 9 | 124614772 | T | G | 0.012 | 0.002 | 2.50E-08 | 36 |
| rs10953765 | 7 | 114291435 | A | G | -0.013 | 0.002 | 4.90E-10 | 42 |
| rs114408770 | 3 | 43873454 | A | G | -0.044 | 0.007 | 1.60E-10 | 40 |
| rs11665242 | 18 | 50907127 | G | A | -0.013 | 0.002 | 2.10E-09 | 42 |
| rs13064576 | 3 | 49642430 | T | C | 0.017 | 0.002 | 2.40E-14 | 72 |
| rs13238996 | 7 | 74069645 | G | A | -0.013 | 0.002 | 3.20E-08 | 42 |
| rs13274119 | 8 | 143342967 | A | C | 0.020 | 0.003 | 5.00E-11 | 44 |
| rs13394374 | 2 | 62263120 | T | C | 0.034 | 0.006 | 3.90E-08 | 32 |
| rs1462163 | 2 | 164333146 | T | C | -0.013 | 0.002 | 1.90E-09 | 42 |
| rs1557341 | 18 | 35127427 | C | A | 0.013 | 0.002 | 3.70E-09 | 42 |
| rs17563464 | 5 | 26913774 | A | C | -0.015 | 0.003 | 3.50E-09 | 25 |
| rs178217 | 14 | 26939332 | C | T | -0.014 | 0.002 | 2.10E-09 | 49 |
| rs2588962 | 10 | 63606492 | A | G | 0.015 | 0.002 | 4.10E-13 | 56 |
| rs2709814 | 4 | 152360870 | C | T | -0.011 | 0.002 | 4.20E-08 | 30 |
| rs2857693 | 6 | 31588384 | T | G | -0.012 | 0.002 | 1.40E-08 | 36 |
| rs34945223 | 18 | 77575871 | G | A | -0.014 | 0.002 | 3.40E-09 | 49 |
| rs4557720 | 8 | 87932823 | G | A | 0.012 | 0.002 | 2.00E-08 | 36 |
| rs4674403 | 2 | 220345117 | G | T | -0.013 | 0.002 | 4.90E-08 | 42 |
| rs4731951 | 7 | 133102282 | T | G | 0.015 | 0.003 | 3.40E-09 | 25 |
| rs55771711 | 10 | 133802737 | C | G | 0.013 | 0.002 | 2.40E-08 | 42 |
| rs57513571 | 17 | 2309130 | T | C | -0.015 | 0.003 | 2.70E-09 | 25 |
| rs62039529 | 16 | 15237487 | A | C | 0.017 | 0.003 | 5.40E-09 | 32 |
| rs6449503 | 5 | 60095272 | A | G | 0.012 | 0.002 | 2.60E-09 | 36 |
| rs6508344 | 18 | 22646735 | A | G | -0.012 | 0.002 | 2.80E-08 | 36 |
| rs6679399 | 1 | 211093346 | C | A | 0.043 | 0.007 | 3.00E-10 | 38 |
| rs6729586 | 2 | 44808475 | A | G | -0.012 | 0.002 | 2.40E-08 | 36 |
| rs68191270 | 1 | 44026180 | C | A | 0.017 | 0.002 | 2.50E-11 | 72 |
| rs6931604 | 6 | 98578215 | T | C | 0.014 | 0.002 | 1.10E-10 | 49 |
| rs7110786 | 11 | 95656648 | T | C | 0.013 | 0.002 | 1.20E-09 | 42 |
| rs7768758 | 6 | 88251077 | C | T | -0.023 | 0.004 | 2.10E-09 | 33 |
| rs7896518 | 10 | 65104500 | G | A | 0.015 | 0.002 | 6.20E-12 | 56 |
| rs7975763 | 12 | 123604053 | T | C | 0.016 | 0.003 | 1.90E-10 | 28 |
| rs9536961 | 13 | 55678332 | G | A | 0.014 | 0.002 | 2.40E-10 | 49 |
| rs9655780 | 7 | 104667334 | A | G | -0.017 | 0.003 | 4.30E-10 | 32 |
| rs9866630 | 3 | 78505146 | A | G | -0.013 | 0.002 | 1.00E-09 | 42 |

SNP, single nucleotide polymorphism; SE, standard error; OA, other allele; EA, effect allele.
