# Supplementary material for: Causal effects of socioeconomic traits on frailty: a Mendelian randomization study
Source: Front Med (Lausanne). 2024 Jul 12;11:1344217. doi: 10.3389/fmed.2024.1344217 (PMC11282504; doi:10.3389/fmed.2024.1344217)
Supplement: Supplementary file 3 [file Table_3.DOCX]

Supplementary Table 3 Characteristics of the instrument SNPs for job involves heavy manual or physical work.

| **SNP** | **Chr** | **Position** | **EA** | **OA** | **Exposure effect** |  |  | **F-statistic** |
| --- | --- | --- | --- | --- | --- | --- | --- | --- |
|  |  |  |  |  | **β** | **SE** | ***P*** |  |
| rs1081158 | 5 | 88004616 | T | C | 0.017 | 0.002 | 2.70E-12 | 72 |
| rs10820625 | 9 | 99203606 | C | T | -0.016 | 0.003 | 2.90E-08 | 28 |
| rs11130889 | 3 | 62562748 | G | C | -0.025 | 0.005 | 4.50E-08 | 25 |
| rs11663824 | 18 | 50745236 | A | C | 0.014 | 0.002 | 2.50E-08 | 49 |
| rs11678979 | 2 | 100802891 | C | T | -0.015 | 0.003 | 3.00E-08 | 25 |
| rs11726786 | 4 | 106120756 | G | T | 0.017 | 0.002 | 2.40E-11 | 72 |
| rs11756123 | 6 | 152218079 | T | A | -0.014 | 0.002 | 3.20E-08 | 49 |
| rs12089815 | 1 | 91189933 | A | G | -0.015 | 0.002 | 3.10E-10 | 56 |
| rs1370059 | 13 | 58523077 | G | A | 0.018 | 0.003 | 1.80E-10 | 36 |
| rs2091329 | 1 | 110042079 | G | A | -0.016 | 0.003 | 2.00E-09 | 28 |
| rs2318540 | 4 | 67831581 | G | T | -0.014 | 0.002 | 3.70E-09 | 49 |
| rs2819348 | 1 | 201884952 | C | T | 0.015 | 0.002 | 5.60E-09 | 56 |
| rs34700731 | 2 | 203360745 | A | G | 0.020 | 0.003 | 1.50E-08 | 44 |
| rs35999162 | 3 | 49597230 | G | C | -0.025 | 0.003 | 7.40E-22 | 69 |
| rs3785354 | 16 | 28582941 | T | C | 0.014 | 0.002 | 3.70E-08 | 49 |
| rs4580876 | 6 | 98322872 | A | G | -0.022 | 0.002 | 3.00E-20 | 121 |
| rs4731992 | 7 | 133702097 | G | A | -0.017 | 0.003 | 4.00E-09 | 32 |
| rs56194430 | 5 | 67824690 | T | C | 0.019 | 0.003 | 4.50E-09 | 40 |
| rs62511803 | 8 | 115992376 | A | G | -0.021 | 0.003 | 7.50E-10 | 49 |
| rs6544763 | 2 | 44813233 | C | T | 0.014 | 0.003 | 3.20E-08 | 22 |
| rs7108077 | 11 | 95838005 | G | A | -0.013 | 0.002 | 4.90E-08 | 42 |
| rs7467480 | 9 | 23354940 | A | T | -0.016 | 0.002 | 1.70E-10 | 64 |
| rs77823953 | 8 | 143524185 | T | C | -0.037 | 0.007 | 3.80E-08 | 28 |
| rs8054111 | 16 | 71990651 | G | A | -0.015 | 0.003 | 1.20E-08 | 25 |

SNP, single nucleotide polymorphism; SE, standard error; OA, other allele; EA, effect allele.
