# Supplementary material for: Causal effects of socioeconomic traits on frailty: a Mendelian randomization study
Source: Front Med (Lausanne). 2024 Jul 12;11:1344217. doi: 10.3389/fmed.2024.1344217 (PMC11282504; doi:10.3389/fmed.2024.1344217)
Supplement: Supplementary file 4 [file Table_4.DOCX]

Supplementary Table 4 Characteristics of the instrument SNPs for job involves mainly walking or standing.

| **SNP** | **Chr** | **Position** | **EA** | **OA** | **Exposure effect** |  |  | **F-statistic** |
| --- | --- | --- | --- | --- | --- | --- | --- | --- |
|  |  |  |  |  | **β** | **SE** | ***P*** |  |
| rs11264886 | 1 | 153997346 | A | G | 0.019 | 0.003 | 1.10E-08 | 40 |
| rs13019832 | 2 | 60710571 | A | G | -0.019 | 0.003 | 1.50E-09 | 40 |
| rs1487445 | 6 | 98565211 | T | C | -0.031 | 0.003 | 6.80E-24 | 107 |
| rs3785354 | 16 | 28582941 | T | C | 0.018 | 0.003 | 7.50E-09 | 36 |
| rs4731992 | 7 | 133702097 | G | A | -0.022 | 0.004 | 1.30E-09 | 30 |
| rs603625 | 11 | 95554283 | A | G | -0.018 | 0.003 | 2.20E-09 | 36 |
| rs613872 | 18 | 53210302 | T | G | 0.024 | 0.004 | 3.90E-09 | 36 |
| rs6603030 | 15 | 83230103 | G | A | -0.021 | 0.004 | 2.40E-08 | 28 |
| rs6882046 | 5 | 87968864 | G | A | -0.025 | 0.003 | 4.90E-13 | 69 |
| rs7661349 | 4 | 106066982 | C | T | -0.020 | 0.003 | 1.60E-10 | 44 |
| rs78928669 | 7 | 71782460 | G | A | -0.048 | 0.008 | 7.70E-09 | 36 |
| rs79248502 | 5 | 111012600 | G | C | -0.038 | 0.006 | 3.10E-09 | 40 |
| rs8054111 | 16 | 71990651 | G | A | -0.021 | 0.003 | 5.30E-10 | 49 |
| rs9341742 | 6 | 79440229 | T | C | 0.017 | 0.003 | 2.00E-08 | 32 |
| rs9836291 | 3 | 49697459 | A | G | -0.027 | 0.003 | 3.30E-16 | 81 |

SNP, single nucleotide polymorphism; SE, standard error; OA, other allele; EA, effect allele.
