# Supplementary material for: Causal effects of socioeconomic traits on frailty: a Mendelian randomization study
Source: Front Med (Lausanne). 2024 Jul 12;11:1344217. doi: 10.3389/fmed.2024.1344217 (PMC11282504; doi:10.3389/fmed.2024.1344217)
Supplement: Supplementary file 5 [file Table_5.DOCX]

Supplementary Table 5 Characteristics of the instrument SNPs for average total household income before tax.

| **SNP** | **Chr** | **Position** | **EA** | **OA** | **Exposure effect** |  |  | **F-statistic** |
| --- | --- | --- | --- | --- | --- | --- | --- | --- |
|  |  |  |  |  | **β** | **SE** | ***P*** |  |
| rs10429582 | 9 | 23346850 | C | T | 0.027 | 0.003 | 7.60E-24 | 81 |
| rs10761035 | 9 | 99236092 | A | G | 0.019 | 0.003 | 3.70E-08 | 40 |
| rs11191116 | 10 | 103555611 | T | C | -0.016 | 0.003 | 3.50E-09 | 28 |
| rs11588857 | 1 | 204587047 | A | G | 0.021 | 0.003 | 4.90E-11 | 49 |
| rs11678501 | 2 | 188919210 | C | T | -0.052 | 0.009 | 1.20E-09 | 33 |
| rs11714337 | 3 | 71582521 | A | G | 0.015 | 0.003 | 9.40E-09 | 25 |
| rs11877758 | 18 | 35138110 | G | T | -0.020 | 0.003 | 1.20E-12 | 44 |
| rs11917431 | 3 | 49644012 | T | C | 0.023 | 0.003 | 3.10E-15 | 59 |
| rs1229984 | 4 | 100239319 | C | T | -0.049 | 0.008 | 1.00E-09 | 38 |
| rs12531825 | 7 | 8005174 | A | G | -0.026 | 0.004 | 2.10E-10 | 42 |
| rs12692596 | 2 | 161265910 | T | C | -0.015 | 0.003 | 1.70E-08 | 25 |
| rs12883788 | 14 | 33303540 | T | C | -0.019 | 0.003 | 1.40E-12 | 40 |
| rs13002946 | 2 | 100801959 | A | T | 0.021 | 0.003 | 7.30E-12 | 49 |
| rs1421334 | 8 | 30865733 | C | A | 0.016 | 0.003 | 1.00E-09 | 28 |
| rs2068428 | 9 | 1792147 | T | C | 0.017 | 0.003 | 4.20E-08 | 32 |
| rs2332719 | 3 | 123712966 | G | A | -0.018 | 0.003 | 5.70E-10 | 36 |
| rs2362523 | 19 | 5002301 | G | A | 0.016 | 0.003 | 1.40E-08 | 28 |
| rs2422859 | 20 | 3132828 | G | T | 0.016 | 0.003 | 6.20E-10 | 28 |
| rs2515919 | 6 | 31564167 | G | A | -0.016 | 0.003 | 5.70E-09 | 28 |
| rs2820314 | 1 | 201872209 | C | A | -0.017 | 0.003 | 2.90E-09 | 32 |
| rs32940 | 5 | 141132286 | C | T | 0.021 | 0.003 | 2.90E-13 | 49 |
| rs34473884 | 10 | 133761285 | A | G | 0.017 | 0.003 | 1.70E-08 | 32 |
| rs387780 | 2 | 32502495 | C | T | 0.017 | 0.003 | 3.30E-09 | 32 |
| rs4115668 | 16 | 28607532 | A | G | -0.018 | 0.003 | 1.10E-10 | 36 |
| rs488786 | 1 | 20888207 | T | C | 0.021 | 0.004 | 6.70E-09 | 28 |
| rs5754738 | 22 | 34280249 | G | A | -0.016 | 0.003 | 2.70E-08 | 28 |
| rs589914 | 11 | 57661032 | G | A | 0.016 | 0.003 | 4.40E-08 | 28 |
| rs6035877 | 20 | 21512532 | C | A | -0.015 | 0.003 | 4.30E-08 | 25 |
| rs62183028 | 2 | 212631483 | T | G | -0.019 | 0.003 | 3.20E-11 | 40 |
| rs6429636 | 1 | 44183540 | T | G | 0.019 | 0.003 | 5.00E-11 | 40 |
| rs6699397 | 1 | 91212216 | G | A | -0.019 | 0.003 | 3.50E-12 | 40 |
| rs6868457 | 5 | 60550041 | C | T | 0.021 | 0.003 | 3.10E-15 | 49 |
| rs73015322 | 6 | 163857445 | T | G | -0.027 | 0.005 | 4.10E-08 | 29 |
| rs75413320 | 19 | 13207284 | C | T | -0.026 | 0.004 | 9.50E-10 | 42 |
| rs7700107 | 4 | 17880416 | C | A | -0.023 | 0.004 | 2.50E-09 | 33 |
| rs77126132 | 7 | 54966738 | A | G | 0.027 | 0.005 | 5.20E-09 | 29 |
| rs784256 | 18 | 53398626 | A | G | -0.025 | 0.003 | 7.90E-14 | 69 |
| rs7896518 | 10 | 65104500 | G | A | 0.015 | 0.003 | 4.10E-08 | 25 |
| rs9388490 | 6 | 126704795 | T | C | 0.015 | 0.003 | 1.50E-08 | 25 |
| rs9556958 | 13 | 99100046 | T | C | -0.015 | 0.003 | 8.50E-09 | 25 |
| rs968050 | 6 | 98574560 | T | C | 0.022 | 0.003 | 3.10E-17 | 54 |
| rs9891103 | 17 | 44091886 | T | C | -0.023 | 0.003 | 9.40E-14 | 59 |

SNP, single nucleotide polymorphism; SE, standard error; OA, other allele; EA, effect allele.
