# Supplementary material for: Causal effects of socioeconomic traits on frailty: a Mendelian randomization study
Source: Front Med (Lausanne). 2024 Jul 12;11:1344217. doi: 10.3389/fmed.2024.1344217 (PMC11282504; doi:10.3389/fmed.2024.1344217)
Supplement: Supplementary file 6 [file Table_6.DOCX]

Supplementary Table 6 Characteristics of the instrument SNPs for Townsend deprivation index at recruitment.

| **SNP** | **Chr** | **Position** | **EA** | **OA** | **Exposure effect** |  |  | **F-statistic** |
| --- | --- | --- | --- | --- | --- | --- | --- | --- |
|  |  |  |  |  | **β** | **SE** | ***P*** |  |
| rs113345285 | 14 | 104246362 | C | T | 0.016 | 0.003 | 3.20E-08 | 28 |
| rs11855821 | 15 | 78008843 | A | G | -0.013 | 0.002 | 3.90E-08 | 42 |
| rs12133063 | 1 | 91214714 | A | C | 0.015 | 0.002 | 6.10E-12 | 56 |
| rs1483246 | 2 | 186050380 | C | T | -0.012 | 0.002 | 3.10E-09 | 36 |
| rs1947083 | 1 | 67004801 | A | G | 0.012 | 0.002 | 1.90E-08 | 36 |
| rs2403326 | 5 | 103791044 | G | A | 0.012 | 0.002 | 1.20E-08 | 36 |
| rs253125 | 5 | 106645685 | C | T | 0.013 | 0.002 | 1.90E-08 | 42 |
| rs3865018 | 15 | 67892766 | T | C | -0.013 | 0.002 | 4.20E-09 | 42 |
| rs4785187 | 16 | 49766772 | A | G | 0.014 | 0.002 | 1.50E-08 | 49 |
| rs56142341 | 1 | 208145995 | C | G | -0.026 | 0.004 | 5.20E-09 | 42 |
| rs62477310 | 7 | 114951541 | C | T | -0.012 | 0.002 | 5.40E-09 | 36 |
| rs6931604 | 6 | 98578215 | T | C | -0.012 | 0.002 | 4.10E-09 | 36 |
| rs704067 | 12 | 89726027 | A | G | 0.012 | 0.002 | 3.90E-09 | 36 |
| rs7740440 | 6 | 142972917 | A | G | 0.012 | 0.002 | 5.70E-09 | 36 |
| rs78257128 | 5 | 164545854 | T | C | -0.017 | 0.003 | 2.40E-09 | 32 |
| rs989532 | 2 | 173933824 | G | A | 0.014 | 0.002 | 7.40E-11 | 49 |
| rs990706 | 11 | 78135704 | T | C | 0.016 | 0.003 | 7.90E-09 | 28 |

SNP, single nucleotide polymorphism; SE, standard error; OA, other allele; EA, effect allele.
