# Supplementary material for: Causal effects of socioeconomic traits on frailty: a Mendelian randomization study
Source: Front Med (Lausanne). 2024 Jul 12;11:1344217. doi: 10.3389/fmed.2024.1344217 (PMC11282504; doi:10.3389/fmed.2024.1344217)
Supplement: Supplementary file 7 [file Table_7.DOCX]

Supplementary Table 7 Characteristics of the instrument SNPs for social isolation/loneliness.

| **SNP** | **Chr** | **Position** | **EA** | **OA** | **Exposure effect** |  |  | **F-statistic** |
| --- | --- | --- | --- | --- | --- | --- | --- | --- |
|  |  |  |  |  | **β** | **SE** | ***P*** |  |
| rs1022688 | 20 | 47648856 | A | G | 0.005 | 0.001 | 1.50E-08 | 25 |
| rs10456089 | 6 | 11959836 | A | G | -0.010 | 0.002 | 2.10E-10 | 25 |
| rs10950394 | 7 | 12263587 | T | C | 0.005 | 0.001 | 2.20E-08 | 25 |
| rs13291079 | 9 | 96360650 | C | T | -0.005 | 0.001 | 2.20E-11 | 25 |
| rs159960 | 1 | 8476428 | G | A | -0.004 | 0.001 | 4.00E-08 | 16 |
| rs2149351 | 9 | 120501644 | G | T | -0.005 | 0.001 | 2.70E-08 | 25 |
| rs30266 | 5 | 103972357 | A | G | 0.005 | 0.001 | 6.70E-09 | 25 |
| rs599550 | 18 | 53252388 | A | G | 0.009 | 0.001 | 3.50E-14 | 81 |
| rs62085660 | 17 | 66097739 | G | C | -0.005 | 0.001 | 5.80E-09 | 25 |
| rs6430286 | 2 | 148834364 | A | G | -0.005 | 0.001 | 1.30E-08 | 25 |
| rs67988891 | 5 | 152204741 | G | C | -0.006 | 0.001 | 1.10E-10 | 36 |
| rs7107356 | 11 | 47676170 | G | A | 0.004 | 0.001 | 3.30E-08 | 16 |
| rs74338595 | 2 | 212749786 | C | T | -0.005 | 0.001 | 4.60E-09 | 25 |
| rs7626596 | 3 | 82000680 | A | G | -0.005 | 0.001 | 5.20E-09 | 25 |
| rs773020 | 9 | 77768122 | A | G | 0.008 | 0.001 | 9.00E-09 | 64 |
| rs7770860 | 6 | 131186393 | C | T | 0.005 | 0.001 | 7.50E-09 | 25 |

SNP, single nucleotide polymorphism; SE, standard error; OA, other allele; EA, effect allele.
